# Circadian rhythms affect bone reconstruction by regulating bone energy metabolism

**DOI:** 10.1186/s12967-021-03068-x

**Published:** 2021-09-27

**Authors:** Beibei Luo, Xin Zhou, Qingming Tang, Ying Yin, Guangxia Feng, Shue Li, Lili Chen

**Affiliations:** 1 Department of Stomatology, Union Hospital, Tongji Medical College, Huazhong University of Science and Technology, Wuhan, 430022 China; 2 School of Stomatology, Tongji Medical College, Huazhong University of Science and Technology, Wuhan, 430030 China; 3 Hubei Province Key Laboratory of Oral and Maxillofacial Development and Regeneration, Wuhan, 430022 China

**Keywords:** Circadian rhythm, Circadian clock gene, Osteogenesis, Bone formation, Skeleton formation, Bone, Metabolism, Osteoclast

## Abstract

Metabolism is one of the most complex cellular biochemical reactions, providing energy and substances for basic activities such as cell growth and proliferation. Early studies have shown that glucose is an important nutrient in osteoblasts. In addition, amino acid metabolism and fat metabolism also play important roles in bone reconstruction. Mammalian circadian clocks regulate the circadian cycles of various physiological functions. In vertebrates, circadian rhythms are mediated by a set of central clock genes: *muscle and brain ARNT like-1* (*Bmal1), muscle and brain ARNT like-2 (Bmal2), circadian rhythmic motion output cycle stagnates (Clock), cryptochrome 1 (Cry1), cryptochrome2 (Cry2), period 1 (Per1), period 2 (Per2), period 3 (Per3)* and *neuronal PAS domain protein 2* (*Npas2)*. Negative feedback loops, controlled at both the transcriptional and posttranslational levels, adjust these clock genes in a diurnal manner. According to the results of studies on circadian transcriptomic studies in several tissues, most rhythmic genes are expressed in a tissue-specific manner and are affected by tissue-specific circadian rhythms. The circadian rhythm regulates several activities, including energy metabolism, feeding time, sleeping, and endocrine and immune functions. It has been reported that the circadian rhythms of mammals are closely related to bone metabolism. In this review, we discuss the regulation of the circadian rhythm/circadian clock gene in osteoblasts/osteoclasts and the energy metabolism of bone, and the relationship between circadian rhythm, bone remodeling, and energy metabolism. We also discuss the therapeutic potential of regulating circadian rhythms or changing energy metabolism on bone development/bone regeneration.

## Introduction

Most organisms, including humans, have a circadian rhythm that exhibits an endogenous oscillation of ~24 h and is synchronized with light/dark cycles through morning food consumption or/and light exposure [1]. Circadian rhythm plays a critical role in most physiological and behavioral processes in mammals. The suprachiasmatic nucleus (SCN) is the controlling center of mammalian rhythm oscillation, the rhythm signals of which are influenced by core clock genes, including *muscle and brain ARNT like-1* (*Bmal1*), *circadian rhythmic motion output cycle stagnates* (*clock*), *cryptochrome* (*Cry*) and *period* (*Per*) and orphan nuclear hormone receptors *Rorα* and *Rev-erbα* [2]. Molecular clocks are known as the mechanisms of circadian rhythms in mammals. They consist mainly of a series of interrelated transcription-translation feedback loops [3]. Temporal information is transmitted by the SCN to peripheral tissue oscillators, producing synchronous circadian rhythms in many bodily processes, including bone metabolism, muscle function, and immune system function [4]. Previous studies have reported that the metabolism of bone and cartilage exhibits circadian rhythms. In an overview of related studies, circadian rhythms can be seen in the expression of the master genes involved in cartilage formation, bone mineral deposition, and bone formation [3, 5–8]. Serum concentrations of some hormones relating to bone metabolism show diurnal variation [9]. For example, serum concentrations of calcium, calcitonin, skeletal alkaline phosphatase, parathyroid hormone C-telopeptide, tartate-resistant acid phosphatase, and osteocalcin show diurnal variation [10–13]. Studies have shown that continuous lighting can lead to a significant decrease in skeletal muscle function and to bone microstructure changes with early osteoporosis characteristics [4]. Studies have shown that chondrocyte-specific *Bmal1* knockout mice exhibit progressive degeneration and damage in knee articular cartilage starting the second month after circadian clock activity of cartilage tissue is disturbed and lasting 3–6 months [14]. Studies have reported that *Per* and *Cry* mutant mice showed increased osteoblast activity and bone mass, while *Bmal1* knockout mice showed ectopic calcification and abnormal cartilage reendothelialization [4].

Bone, a metabolically active organ, undergoes continuous remodeling due to bone formation by osteoblasts and bone absorption by osteoclasts [15]. In healthy situations, the balance between bone absorption and bone formation is consistent, maintaining bone density and bone strength. Some pathological conditions can affect bone reconstruction, which can lead to bone disease [16]. Bone remodeling requires energy, and growth and repair after bone damage requires more energy [17]. For example, bone healing is the main prognostic factor of oral maxillofacial surgery, and adequate nutrition plays a vital role in fracture repair. Severely malnourished patients show slow wound healing and damage to wound contraction [18]. Studies have shown that oscillations in circadian rhythms lead to rhythmic changes involving physiological processes such as nutrition and metabolism [19]. Moreover, studies have shown that nutritional deficiencies in children are often associated with developmental impairment, which can affect normal bone growth and development. Some defects also directly affect cartilage and bone production [20]. The circadian rhythms of bone functions are regulated by internal or external cues. Feeding and fasting regulate the daily rhythm of the bone turnover marker serum C-telopeptide fragments of collagen type 1 degradation (S-CTX) [21]. The levels of S-CTX are higher in humans during early morning, from 05:00 to 08:00, and lower in the late afternoon, from 12:00 to 16:00. S-CTX have a clear daily rhythm across the 24 h day under normal feeding conditions, such as the consumption of breakfast, lunch and dinner, while the amplitude of the rhythms is diminished with fasting [22, 23]. In addition, the generation of this diurnal variation is also observed in intake of glucose, protein, and fat [22]. Circadian disruption can occur with social or environmental factors, such as shift work, may cause dysfunctions of bone and skeletal muscle. In epidemiological studies, the prevalence of metabolic syndrome, bone fractures and osteoporosis are increased in shift workers [24–26]. These findings indicate that the regulation of circadian rhythms in bone by external cues, are important for the maintenance of homeostasis. The interactions involved in bone and energy metabolism are mediated by a variety of nutrients, hormones, and cytokines [27]. Therefore, we explore new ways for the circadian clock to regulate energy metabolism and promote bone development/bone regeneration, and we suggest new strategies for bone reconstruction.

## Circadian rhythm in bone

**Fig. 1:**
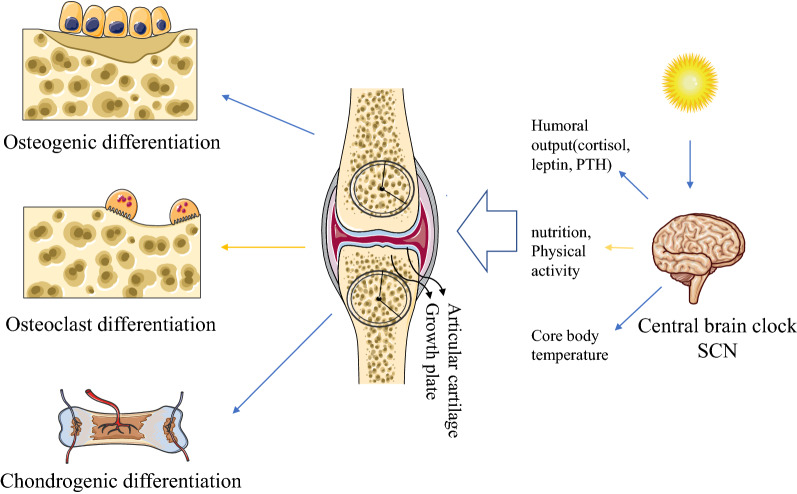
A pattern diagram of the circadian rhythm regulation of bone

Currently, due to light pollution at night, passive or active wakefulness late into the night, night shift work and other reasons, the body’s circadian rhythm is greatly affected. Studies have shown that circadian rhythm disorders are causes of diminished bone microstructure [28]. Studies have shown that circadian rhythms can affect the development and growth of the mandible; however, to a large extent, circadian dysrhythmia inhibits the growth of the mandible [29]. Continuous light exposure can lead to trabecular bone deterioration and induce a short-term inflammatory state [4]. Epidemiological studies have shown that the incidence of osteoporosis and fractures among shift workers is high [4]. IL-6, an important pro-inflammatory cytokine expressed in rheumatoid arthritis (OA) [30], mediates osteoclast activation, thus contributing to cartilage and bone breakdown and joint destruction. Previous studies have found that plasma IL-6 levels in rats with collagen-induced arthritis (CIA) were higher at almost every sampling time compared to those in normal rats, and IL-6 expression showed significant circadian rhythms, indicating higher levels during the light phase and lower levels during the dark phase [31]. Recent studies revealed a relationship between circadian rhythm and osteogenesis and osteoclastogenesis. The study found that under the effect of the peripheral nervous system, bone-related genes and bone absorption-related genes show periodic expression patterns [32]. Circadian time cues in bone affect osteoblast and osteoclast differentiation, and bone transformation markers exhibit circadian changes [33] (Fig. 1). The expression of parathyroid hormone (PTH), C-telopeptide of type 1 collage (CTX), calcium, bone specific alkaline phosphatase (BSAP), and calcitonin exhibits circadian rhythm changes [9]. In osteoblasts, 26% of genes show daytime expression patterns, including bone morphogenetic protein 2 (*Bmp2*), insulin-like growth factor 1 (*Igf1*), osteocalcin (Oc, *Bglap*), and the major osteoblast transcription factor Runt-related transcription factor 2 (*Runx2*). In humans, circadian rhythms coordinate bone remodeling [34]. The circadian clock is related to bone development and the regulation of homeostasis in bone, molecules associated with osteoblast differentiation are controlled by circadian rhythms, and genes including dentin matrix protein 1(*Dmp1*), osteopontin(*Spp1*), bone sialoprotein(*Bsp*)and osteocalcin(*Bglap2*) involved in mineral deposition are expressed in circadian rhythm patterns [5, 35–37].

Bones are tissues that continue to be remodeled, and circadian rhythm disorders have a negative impact on bone health. Studies have shown that the circadian clock gene is related to bone formation [38]. *Bmal1* plays a key role in regulating bone absorption and bone formation and is the core and irreplaceable component of the circadian rhythm molecular oscillator [39]. The overall absence of *Bmal1* in mice led to a decrease in bone mass, which contributed to an increase in bone absorption [40]. Osteoclast-specific *Bmal1* knockout mice showed high bone mass-related pelage and additional bone formation compared to wild-type mice because of reduced osteoclast differentiation [9, 40]. Studies have shown that *Bmal1* controls bone absorption by upregulating the expression of nuclear factor of activated T cells, cytoplasmic, calcineurin-dependent 1 (*Nfatc1*) transcription through its binding to an E-box element located in the *Nfatc1* promoter and interacting with members of the steroid receptor coactivator family [41]. Studies have reported that the clock system also exists in osteoblasts [38]. Coculture experiments have shown that osteoblasts with *Bmal1* defects exhibit a strong ability to induce the differentiation of osteoclasts, while overexpression of *Bmal1/Clock* inhibits the expression of calcitriol-induced receptor activator of NF κB ligand *(Rankl)*, in osteoblasts [40]. Bone homeostasis is affected by circadian rhythms, with bone absorption increased at night compared to daytime. Bone density in the alveolar septum and cortex of the mandible was lower in *Bmal1*^*−/−*^ mice than in wild-type mice [42]. Moreover, the *Bmal1*^*−/−*^ animals form short bones and present with osteopenia in the mandibular condyle and long bones [29]. The absence of *Bmal1* promotes the differentiation of osteoclasts, inhibits the differentiation of osteoblasts and cartilage cells, and ultimately leads to a decrease in bone mass and bone density. Studies have found that osteogenic differentiation is inhibited in bone marrow stromal cells (BMSCs) and that femoral bone mass is obviously reduced in *Bmal1*^−/−^ mice [42]. *Bmal1* can also affect myelin cells, regulate the bone mass of the discs between vertebrae and is closely related to the height of these discs [43]. The study revealed a relationship between circadian rhythm and the development of mineralized tissue, including osteoclastogenesis and osteogenesis [44, 45]. In addition to *Bmal1*, *Clock*, *Cry* and *Per* are also the main genes of the molecular clocks. Recent studies also showed that *Cry2* and *Per2* affect multiple mechanisms of adjusting bone volume [32]. *Cry2* affects osteoclast activity [46]. In addition, *Cry2* plays an active role in the steady state of the extracellular matrix of cartilage [47]. *Per2*, a negative regulation factor of circadian clock, is necessary for the maturation of bone tissue. *Per2* mutation increases the proliferative ability of osteoblasts [48]. The bone volume was increased in *Per* and *Cry* deficient mice [42].The bone density of mice with mutated *Clock* was significantly decreased, and the apoptosis rate was increased [38].

Joint cartilage arranged on the surface of long bones is a highly specialized connective tissue. Emerging evidence suggests that circadian rhythm systems play key roles in controlling bone biology and cartilage [13]. Most core clock genes are rhythmically expressed in different types of cartilage, including sword cartilage, facial joint cartilage, growth plates, and rib cartilage [49, 50]. It has been reported that 619 genes (3.9% of expressed genes) exhibit circadian rhythm expression patterns in cartilage, including genes that are involved in the stability and survival in cartilage, as well as genes that are potentially important in the pathogenesis of osteoarthritis (OA) [51]. Studies have shown that the most active proliferation stages, which were found by measuring cartilage cell proliferation markers, are in the early morning, causing growth plate expansion to peak at noon [52]. The circadian rhythms of living cartilage can be system-driven, similar to that in other tissues, or by local molecular clocks in cartilage cells [53]. Long-term (22-week) environmental disturbances affect the dark/light cycle and thus the circadian rhythms in mice, generating a condition similar to years of chronic jet lag or shift work, making mouse knees susceptible to OA-like injuries [54]. *Bmal1* controls the dynamic balance and integrity of cartilage [6]. In a mouse model of OA, several clock genes in the early stages of cartilage degeneration were disrupted, suggesting a role for circadian rhythms in maintaining a steady state in cartilage. Changing the light and dark cycle of the environment to interfere with circadian rhythms contributed to changes that induce knee osteoarthritis in mice [51].

When it comes to bones, skeletal muscles will be mentioned. Studies have shown that mechanical loads and endocrine factors may be the way for bone and muscles communicating with each other [55]. Studies have shown that skeletal muscle molecular clock are associated with skeletal homeostasis. Loss of *Bmal1* solely from adult skeletal muscle (iMS*Bmal1* ^−/−^) results in severe skeletal system pathology, similar to that observed in the *Bmal1* knockout mice. iMS*Bmal1* ^−/−^ have the appearance of misshapen tibia and fibula, flattened tarsals, and increased calcification throughout the rib cage and thoracic spine, which was related to changes in the endocrine/paracrine function of muscle [56]. Therefore, endogenous skeletal muscle molecular clock is a modulator of musculoskeletal health.

## Energy metabolism (glucose, amino acid, and fat) in bone

### Glucose metabolism in bone

**Fig. 2:**
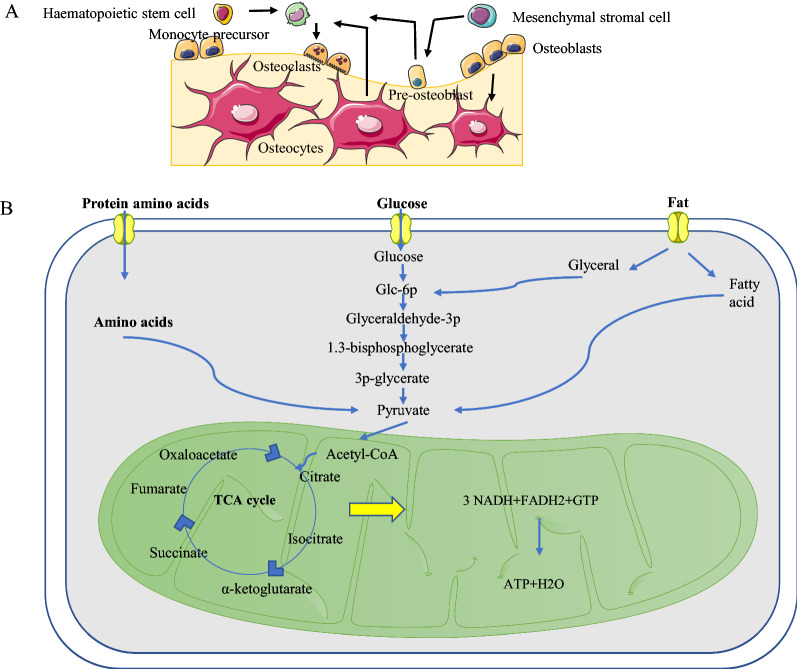
**A** Patterns of osteoblast and osteoclast differentiation. **B** Pattern diagram of energy substrates and intermediate metabolic pathways

In bone systems, glucose is a necessary source of energy for bone and joint cartilage development, growth and maintenance (Fig. 2). During embryo growth and fetal development, bone morphology is particularly important [57]. High blood sugar levels inhibit calcium absorption and bone calcification [58]. Increasing evidence suggests that bone metabolism is closely related to glucose metabolism [59]. Early studies of bone transplantation or isolated osteoblastic cells have shown that glucose is an important nutrient in osteoblasts [60]. Bone absorption has been shown to rely on glycolysis. Studies have shown that osteoclasts fueled by galactose significantly reduced the degradation of type 1 collagen by reducing the rate of glycolysis, forcing cells to rely on oxidative phosphorylation [61]. One study confirmed that glucose intake by osteoblast precursors was the earliest determining factor in osteoblast differentiation and bone formation [62]. Disorder of glucose metabolism changes the maturation process of cartilage cells, suggesting that glucose metabolism plays an indispensable role in the process of bone formation in cartilage. Glucose metabolic disorders are likely not only associated with abnormal cartilage growth but also the force driving these changes [63].Abnormal bone metabolism is a typical phenomenon in diabetic patients [27]. In studies, the bone density and bone strength of the lower limbs were significantly reduced in type 2 diabetes mellitus (T2DM) mice, serum osteocalcin levels were significantly reduced, and serum tartrate-resistant acid phosphatase-5p (TRAP) levels were significantly increased, indicating that bone brittleness in T2DM mice was due to increased bone absorption and decreased bone formation [15, 64]. Therefore, we can know that glucose metabolism abnormality is very harmful to bone health, energy metabolism is of great significance to bone development and bone regeneration [65]. Studies have shown that bone cells, dysfunction of osteoblasts and collagen crosslinking induced by advanced glycation end products (AGEs) are associated with bone brittleness in diabetes. Increased levels of homocysteine and AGEs in the circulation of diabetic patients directly impair the function of osteoblasts and other bone cells, leading to reduced bone formation and bone remodeling. When the bone reconstruction process is disrupted, old bone tissue is not renewed and AGE-induced collagen crosslinking is not re-established, leading to a deterioration in bone quality. High blood sugar and AGEs directly or indirectly inhibit bone formation and differentiation of osteoblasts by increasing the level of sclerosis of bone cells and by reducing *Rankl* expression in osteocytes, reducing the degree of bone reconstruction [59]. It is thought that high sugar levels inhibit the differentiation of osteoblast precursors and osteoblasts [66, 67]. In addition, high concentrations of glucose can lead to excessive production of AGEs and reactive oxygen species (ROS) [64], resulting in less substantial mineralization and abnormal bone formation [68]. Osteocalcin has long been considered a marker of new bone production and is now considered the first hormone produced by bones. Serum osteocalcin levels were found to be associated with new bone formation and the number of osteoblasts [69]. Osteocalcin is a non-collagenous protein secreted by osteoblasts and odontoblasts in the final stages of differentiation and an important factor in mineralization, and its level reflects the degree of bone remodeling [17]. Therefore, bone can be considered closely related to glucose metabolism [59]. Studies have shown that glucose-dependent insulinotropic polypeptide (GIP) and glucagon-like peptide-1 (GLP-1) play important roles in the stable state of glucose metabolism and may be associated with the regulation of bone metabolism [15]. Previous studies have reported that increased glycolysis is involved in osteoblast differentiation caused by Wnt signaling. Studies have shown that Wnt7b increases the expression of glucose transporter 1 (Glut1) and glucose consumption in primary cultures of osteoblasts, while the absence of Glut1 inhibits the in vitro differentiation of osteoblasts. Increased glycolysis mediates bone formation induced by Wnt7b [70]. Studies have revealed a link between glycolysis and osteoblast differentiation [71]. Thyroid side gland-produced hormones and bone-forming Wnt proteins such as Wnt3a and Wnt10b stimulate aerobic glycolysis of osteoblasts [72, 73]. Runt-related transcription factor 2, a crucial transcription factor for osteoblast differentiation, induces the expression of Glut1 in osteoblasts, while glucose intake inhibits the degradation of Runx2 to promote osteoblast differentiation [62]. Therefore, the study supports the supposition that increased glucose metabolism is an important mechanism for osteoblast differentiation and function [70]. Glucose and fatty acid metabolism are associated with bone anabolism in response to Wnt signaling [74]. Osteoblasts are derived from mesenchymal stem cells (MSCs) and are also ancestors of fat cells. Osteoclasts are derived from hematopoietic stem cells [75]. When glucose metabolism is impaired, peroxisome proliferative activated receptor gamma (PPARγ) causes an increase in bone transformation by shifting inter-bone marrow-filled stem cells into fat cells [75]. A recent research shows that a history of night shift work and unhealthy lifestyle can independently or synergistically increase the risk of T2DM [76]. Data show that impairment of glucose metabolism is an emerging risk factor for T2DM, which often occurs in circadian rhythm disorders and sleep deprivation caused by shift work [77]. During shift work, due to circadian rhythm disturbance and sleep deprivation, energy expenditure is affected and energy intake increases, leading to overweight and obesity, which in turn increases the risk of T2DM [78]. Study have shown that the circadian rhythm of bone resorption is regulated and maintained by the feeding and fasting rhythm, although the preventive effect of food intake on bone resorption remains to be studied in depth [79].

### Amino acid metabolism in bone

Studies have shown that amino acid restriction alters bone growth and bone differentiation in rodents [80–83]. Homocysteine, formed by methionine demethylation, is a sulfur-containing amino acid. Hyperhomocysteinemia independent of bone density increases the risk of osteoporotic fractures [84]. In summary, methionine is converted into *S*-adenosylmethionine (SAM), which is a common methyl group donor that contributes to the synthesis of homocysteine [85]. Studies have shown that increases in homocysteine increase the apoptosis rate of osteoblast-derived cells, such as osteocytes [86], osteoblasts [87], and bone marrow stromal cells [88]. Homocysteine can then be re-methylated or vulcanized. After this reaction, glutathione and cysteine are synthesized, which play important roles in antioxidant capacity and protein synthesis, respectively [85]. l-arginine (Arg) and l-lysine (Lys) are essential amino acids in the body and are closely related to osteoporosis and bone and defects [89]. Changes in crosslinking and hydroxy lysine level are related to the mechanical capacity of bones [90, 91]. The content of lysine in collagen plays an indispensable role in the composition of collagen fibred crosslinking. The crosslinking process of bone collagen is essential for bone substation formation and is considered important in bone reconstruction [90] and fracture healing. Bone conversion markers, type I pre-collagen N-end peptides and type I collagen C-end peptides, such as provide sensitive indicators of bone formation and bone absorption, respectively [92]. Metabolomics-based studies have reported correlations found with amino acid and bone health assessments [93–95]. In a cross-sectional study of women aged 18–79 years, studies linked arginine, alanine, glutamate and proline intake to higher bone density in the forearms and spine [96]. In addition to the potential impact on collagen synthesis, arginine also promotes the production of bone cells. Tryptophan metabolism is thought to affect the activity of osteoclasts [89, 97, 98]. Mechanistically, surface molecules on T cells (CTLA-4) combined with CD80/86 can activate the enzyme indoleamine 2,3-dioxygenase (IDO) in osteoclast precursors, thereby degrading tryptophan and promoting apoptosis [99].

### Fat metabolism in bone

There is a close link between bone and fat metabolism [100]. The bone trabecular area is where the active bone remodeling process takes place. The positioning of bone fat in the bone trabecular area suggests that bone marrow fat may be involved in bone reconstruction, possibly by providing energy for hematopoietic and bone marrow filling [101]. Fat cells play key roles in maintaining energy balance, storing energy in the form of lipids and releasing fatty acids when metabolic signals or energy is low [102]. Previous studies have reported that adipose tissue is related to bone metabolism. Organ fat cells not only store energy but also secrete a variety of bioactive factors called fat factors [59]. Studies have shown that there is an inverse relationship between bone mass and fat mass in bones. The increase in fat content in bone was found to be negatively related to bone mass in ageing and negatively related to the decrease in bone acquisition during growth [103–105]. The peroxisome proliferator-activated receptor 2 (PPAR-2) subtype is activated with natural (fatty acids and ecological steroids) or artificial (TZD) ligands to induce bone marrow mesenchymal stem cells to differentiate into fat cells at the expense of bone-forming-cell development, resulting in bone mass reduction [106–108]. Obese patients usually exhibit higher bone mineral density than people of normal weight [109–111]. Endocrine activity in fat cells leads to the production of fat factors, through which leptin and lipids regulate the calorie intake and insulin sensitivity of the outer tissue, respectively; these fat factors are also produced in bones [101]. The main function of leptin is to regulate the amount of fat stored in the body, which regulates hunger and energy consumption [112]. Leptin was the first hormone found in adipose tissue more than 20 years ago, and since its discovery, evidence has accumulated to support the hypothesis that adipose tissue is an endocrine organ associated with regulating energy metabolism [113]. Studies have shown that adiponectin inhibits the formation of fat cells, stimulates the phenotype acquisition and cell proliferation of osteoblasts, and inhibits the production of osteoclasts in vitro [114]. Animal studies have shown that leptin increases bone density, bone mineral content and bone formation [115, 116]. Studies have shown that Wnt, PTH and IGF signaling stimulates glycolysis and glutamine to induce metabolism while increasing bone accumulation [117]. Leptin, a fat cell-derived hormone, regulates bone metabolism through the sensory nervous system and the central nervous system [118]. Fat levels have been found to be associated with bone density and fracture risk [119]. Studies in animals have shown that fat-muscle-bone relationships may be associated with circulating osteocalcin, a bone cell- and osteoblast-specific peptide [120].

## The circadian clock regulates energy metabolism

The circadian rhythm regulates several activities, including energy metabolism, feeding time, sleep, and endocrine and immune function [147]. In recent years, a great number of studies have emphasized that the circadian clock system is closely related to the maintenance of energy metabolism [148–153] (Table 1). The circadian clock has been shown to regulate the daily fluctuations of certain human metabolites, such as glucose [154] amino acids and fatty acids, regardless of the fasting/eating cycle [155]. These metabolites control and regulate physiological processes at the cell, organ and biological levels, integrating signals received from outside the cell and generated during normal metabolism with different control mechanisms to accommodate possible local disturbances while maintaining circadian rhythms, optimizing energy distribution in most cases [147]. Transcription studies have shown that many genes involved in biosynthetic and metabolic processes are rhythmic and that their expression changes throughout the physiological cycle [156–159]. A 48-h assessment of human plasma samples revealed that most metabolites (109 clock metabolites of 171 metabolites) oscillated during a full 24-h waking/sleep cycle [160]. Thus, for humans, most circulating metabolites exhibit rhythmic circadian oscillations under normal physiological conditions [161].

**Table 1:** Metabolic phenotypes of clock disorders in mice

Mouse/experiment/mutation	Phenotype/mechanism	References
Chronic jet lag (6 h advance/week)	Weight gain	[121]
SCN lesion	Loss of behavioral and molecular rhythms, obesity, hyperphagy	[122]
Light exposure at night	Metabolic and behavioral phase shifts, weight gain	[123, 124]
*Per3* knockout	Increased fat mass	[125]
Liver *Bmal1* knockout	Hypoglycemia during fasting period	[126]
Pancreas *Bmal1* knockout	Hypoinsulinemia	[127]
*Cry1/2* double knockout	Loss of behavioral rhythms, hyperinsulinemia	[126, 128]
global *Bmal1* knockout	cataract, sarcopenia, arthropathy, and so on	[129]
*Clock Δ19* mice and the *Clock/Npas2* double knockout	spontaneously calcifying tendons	[130]
*Cry*	CRY also inhibits the transmission of signals downstream of glucagon receptors, thus affecting the production of glycosomes at specific time	[131]
REV-ERB and HNF6	REV-ERB and HNF6 interact to regulate lipid metabolism	[132]
*Npas2*^*−/−*^ mice	Lack nap-type rest periods during the activity Period and cannot be adjusted properly when the eating Time suddenly changes	[133]
Global Per1/2 *knockout*	Reduced total hepatic triglycerides lever	[134]
*Clock, Bmal1, Cry2*	Single nucleotide polymorphisms in *Cry2*, *Bmal1* and *Clock* can alter an individual's risk of type 2 diabetes, abnormal blood lipids	[135–137]
*Clock* mutant, *Bmal1*^*−/−*^ and *Rev-erbα*^*−/−*^ mice	Hyperlipidemic Age-related skeletal muscle loss Fiber-type shift Impaired muscle regeneration	[129, 138, 139]
liver-specific *Bmal1* or *Rev-erbα* deletion	Levels of triglycerides, cholesterol and free fatty acids increased during circulation	[140, 141]
*Global Clock Δ 19 *mutation	Decreased glucose tolerance. Reduced plasma free fatty acids	[142]
*Clock* mutant	lack the circadian pattern of enterocyte gene expression and lipid absorption The disruption of myofiber architecture Reduction in muscle strength. Reduction in mitochondria	[143]
Muscle-specific *Bmal1* knockout	Insulin resistance and glucose intolerance Impaired insulin stimulated glucose uptake Increased muscle mass and size Decreased muscle strength	[144]
*Per2* knockout or *Per2* mutant	No change in muscle mass and lower exercise tolerance	[8]
*Rev-erbaα* knockout	Disruption of myofiber architecture Lower exercise capacity Slight fiber-type shift	[145, 146]

**Fig. 3:**
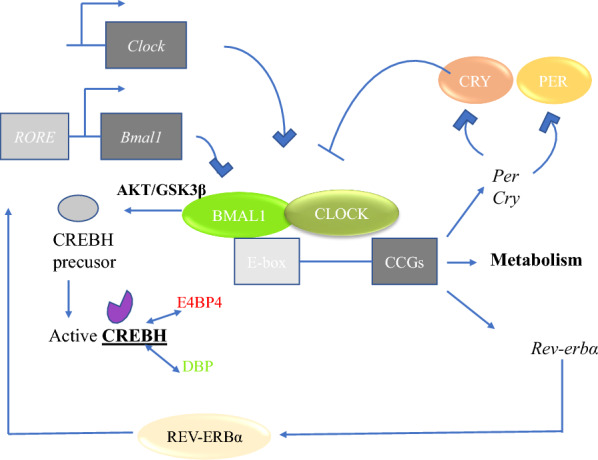
The core molecular circadian clock is present in all mammalian cells and consists of a single unit of positive (CLOCK and BMAL1) and negative (CRY, PER, and REV-ERB alpha). Molecular clocks regulate the expression of hundreds of clock control genes (CCGs), including metabolic media. BMAL1 regulates the activation/cracking of CREBH via AKT-GSK3β signaling. Core circadian oscillations regulate CREBH activity: (1) The core circadian oscillation BMAL1 protein regulates CREBH protein hydrolytic activation; (2) the output circadian rhythm regulator DBP or E4BP4 interacts with the activated CREBH protein to inhibit or synergize with CREBH activity

Rhythm disorders lead to a decline in quality of life and are involved in metabolic syndrome, the development of obesity and neuro-psychiatric disorders [147]. Work associated with circadian rhythm disorders is shift work and air travel across meridians (jet lag). Shift work can disrupt central and outer biological clocks synchronization, further disrupting glucose metabolism by reducing insulin sensitivity, independent of sleep loss [162, 163]. Under dynamic equilibrium conditions, the clock rhythm is the driving force of biological metabolism [164]. Glucose metabolism is a complex physiological process. In humans, daily changes in insulin sensitivity and insulin secretion within 24 h were shown to fluctuate according to significant daily rhythms [165]. Studies have shown that the circadian clock system may maintain a dynamic balance of sugar metabolism by regulating the activity of key enzymes in glucose metabolism [166]. *Pax6* mutant mice (*Pax6*^*Leca2*^) have disorganized melanin-positive intrinsically photosensitive retinal ganglionic cells in eye-like structures and show loss of circadian rhythm. In vivo studies have shown that *Pax6*^*Leca2*^ mice had reduced liver glucose production and reduced hepatic function, possibly due to a loss of rhythm in the metabolic process [167]. In mice, the absence of liver-specific *Bmal1* led to reduced liver glucose production and increased glucose tolerance [168]. Therefore, as observed in the *Pax6*^*Leca2*^ mice, the loss of circadian rhythms may result in inhibition of liver function [167]. In addition, the loss of *Bmal1* and *Clock* may lead to increased insulin sensitivity, which may be due to interruption to the core clock composition in living individuals [165, 168, 169]. At the cellular level, mitochondrial redox reactions [170], phosphate oxide [171, 172], and antioxidant defences [173] are regulated not only by circadian rhythms but also by feedback signaling to the core clock. Studies by Ashley et al. have shown that models of multifunctional amino acid-substituted cells obtained from patients with Snyder-Robinson syndrome indicate that mitochondrial dysfunction is a potential cause of bone defects [174]. Studies by T aira Wada et al. have shown that *Bmal1* regulation of skeletal muscle metabolism provides more insight into the link between obesity/diabetes and the circadian clock system in energy metabolism [175]. Studies by Ik Dong Yoo et al. have shown that overexpression of *Clock* and *Bmal1* significantly inhibited aerobic sulfation and lactic acid production by reducing the protein expression levels of hexokinase 1 (HK1) and lactate dehydrogenase A (LDHA) [176]. Redox stress is an important metabolic regulation factor [177]. Circadian rhythm disorders alter a variety of proteins that are known to regulate glucose stability and/or energy metabolism and are involved in changes in metabolic physiology. The findings of Christopher M. Depner et al. demonstrated that studying the circadian clock, behavioral food intake-fasting/waking-sleep cycles, and the interactions between these processes help in identifying mechanisms that can lead to metabolic disorders and regulate the 24-h pattern in human plasma protein expression [178]. Many of the rate-limiting steps in metabolic pathways associated with metabolic diseases are regulated by circadian rhythms [179], further suggesting circadian rhythm disorder roles in metabolic disorders. Eun Roh et al. found that clock genes mediate the regulation of neuropeptide Y (NPY) and agouti-related protein (AGRP) transcription and suggested a new mechanism explaining the association between clock genes and system metabolic regulation. Overexpressed nicotinamide adenine dinucleotide (NAD) may help obese mice restore day and night fluctuations in retarded metabolic behaviors by enhancing the interaction of clock genes with NPY and AGRP [180]. CREB, hepatocyte specific (CREBH) regulates the acute stage response and energy balance of the liver under stress conditions and is regulated by the circadian clock [170]. Circadian rhythms are produced through a network of clock-controlled genes at the level of gene transcription that form an automatically regulated feedback loop [181]. The *Clock/Bmal1* heterogenic dimer drives the circadian rhythm expression of many other transcription factors, enhancing and extending other circadian rhythm- adjusted functions. Liver nuclear targets or transcriptional regulators may be direct links between circadian rhythms and metabolic pathways [175]. Ze Zheng et al. showed that CREBH activation is regulated by circadian oscillations in the liver and that CREBH is an organ-specific circadian rhythm regulator of lipid metabolism. The dysfunction of CREBH led to impaired rhythms of triglyceride and fatty acid expression. The study found that the core circadian oscillator *Bmal1* regulated the activation/cracking of CREBH via AKT-GSK3β signaling (Fig. 3). Core circadian oscillations regulate CREBH activity: (1) Core circadian oscillation *Bmal1* regulates CREBH protein hydrolytic activation; (2) the output circadian rhythm regulator DBP or E4BP4 interacts with the activated CREBH protein to inhibit or synergize with CREBH activity [182]. In a two-year study, Leticia Goni and others found a significant interaction between the melatonin receptor 1B (MTNR1B) gene and dietary fat intake, affecting changes in obesity level, body composition and fat distribution [183]. The role of circadian rhythm systems in controlling energy balance has long been thought to be associated with the regulation of body fat [184]. Circadian Clock-mutant mice showed a decrease in transcript expression of specific hypothalamic peptides encoded to participate in energy balance [183]. Mice fed a high-fat diet with a mutation in circadian clock developed obesity at a young age, as well as metabolic and endocrine abnormalities consistent with metabolic syndrome [129]. Studies have shown that the transcription factor co-activation by PGC-1a is an important function of the liver and muscle circadian clock and is the main regulator of mitochondrial biological occurrence and energy metabolism. There is a regulatory circuit between the clock machinery and metabolism [161]. The fat cell-derived hormone leptin plays a vital role in metabolic control by reducing food intake and increasing energy consumption. Studies by Alisa Boucsein et al. show that leptin sensitivity is controlled within a 24-h rhythm, in which diet-induced obesity (DIO) is disrupted, causing impaired energy metabolic regulation [185]. Feeney et al. reported a novel physiological function of Mg^2+^ in cell circadian rhythm regulation. They observed rhythmic changes in the concentration of magnesium ions in cells that regulate cell timing and energy balance. Mg^2 +^ is an important rate-limiting factor for many metabolic effects; therefore, we think that the differential expression level of PRL2 (a member of the phosphatase family expressed in regenerating liver) can regulate the concentration of Mg^2+^ in cells to balance the energy needs of cells. The results of noriko Uetani et al. suggest a model in which the daily oscillations caused by PRL2 expression produce rhythmic Mg^2+^ currents, which lead to an appropriate daily metabolic cycle [186].

## Circadian rhythm and bone energy metabolism

Circadian rhythms regulate activities such as eating time, sleep, energy metabolism, and endocrine-related case conditions under the action of a circadian clock located in the central nervous system and peripheral cells [187]. The study also found an interesting phenomenon in which methotrexate raises the important cell circadian rhythm gene, leading to the apoptosis of sliding membrane fibroblasts. The link between circadian rhythms of the disease and time therapy for rheumatoid arthritis is promising [188, 189]. Bone reconstruction is a continuous process of bone formation by osteoblasts and bone absorption by osteoclasts to maintain balance [190]. The development and differentiation of these two different cells are strictly regulated by many endogenous substances, including growth factors, hormones, cytokines and neurotransmitters [191]. Glucose is the main source of energy for most mammalian cells. Glucose is metabolized in the cytoplasm through glycolysis. Glycolysis produces many intermediate metabolites that are essential for various biosynthetic pathways. Pyruvate, the final product of glycolysis, can be converted into lactic acid or further oxidized in the tricarboxylic cycle [60]. The intermediates of tricarboxylic acid are usually extracted from the cycle through a process called quenching to support biosynthesis, redox regulation and the apparent genetic regulation of lipids and amino acids [192–194]. There is growing evidence to show that glycolysis in osteoblast cells is directly stimulated by a variety of anabolic signaling pathways in bone [60]. Parathyroid hormone signaling was previously shown to stimulate lactic acid production and glucose consumption in bone implants before it was clinically used to promote bone formation in osteoporotic patients [195]. The activity of osteoblasts and osteoclasts is regulated by a series of signaling pathways, including parathyroid hormone signaling pathways, and more importantly, parathyroid hormones are expressed via circadian rhythms [190]. In addition to hormones, other factors that regulate bone reconstruction are also rhythmically expressed, such as calcium, osteocalcin, C-telopeptide, bone alkaline phosphatase, and calcitonin [10–12]. So we can make a bold guess that circadian rhythms can affect bone building by regulating the energy metabolism of the bone.

## Summary and outlook

The circadian clock is associated with bone development and the regulation of bone homeostasis; most molecules involved in osteoblast differentiation are controlled by circadian rhythms, and most genes associated with mineral deposition appear in circadian rhythm patterns [5, 35–37]. In bone systems, glucose is a necessary source of energy for bone and joint cartilage development, growth and maintenance. During embryo growth and fetal development, bone morphology is particularly important [57]. At the same time, amino acid metabolism and fat metabolism also play important roles in bone differentiation [117, 196]. Therefore, energy metabolism is of great significance for bone development/bone regeneration. In recent years, many studies have emphasized that the circadian clock system is closely related to the maintenance of energy metabolism [148–153]. These reports show that circadian rhythms can regulate bone activity, which is important for bone development/bone regeneration, and that the circadian clock regulates energy metabolism. However, the relationship between circadian rhythms and bone energy metabolism has not been well articulated, and further evidence is needed to advance the understanding of the interaction between the circadian clock and bone energy metabolism.

## Data Availability

Not applicable.

## Abbreviations

SCN: Suprachiasmatic nucleus
*Bmal1*: Muscle and brain ARNT like-1
*Clock*: Circadian rhythmic motion output cycle stagnates
*Cry*: Cryptochrome
*Per*: Period
Npas2: Neuronal PAS domain protein 2
S-CTX: Serum C-telopeptide fragments of collagen type 1 degradation
OA: Rheumatoid arthritis
CIA: Collagen-induced arthritis
PTH: Parathyroid hormone
CTX: C-telopeptide of type 1 collage
BSAP: Bone specific alkaline phosphatase
Runx2: Runt-related transcriptional factor 2
Dmp1: Dentin matrix protein 1
*Bsp*: Bone sialoprotein
Igf1: Insulin-like growth factor 1
Oc: Osteocalcin
Bmp2: Bone morphogenetic protein 2
NF: Nuclear factor
Nfatc1: Nuclear factor of activated T cells
Cytoplasmic: Calcineurin-dependent 1
RANKL: Receptor activator of NF-κB ligand
BMSCs: Bone marrow stromal cells
SMH: Skeletal mandibular hypoplasia
AGEs: Advanced glycation end products
ROS: Reactive oxygen species
OCN: Osteocalcin
GIP: Glucose-dependent insulinotropic polypeptide
GLP-1: Glucagon-like peptide 1
Glut1: Glucose transporter 1
MSC: Mesenchymal stem cells
PPARγ: Peroxisome proliferative activated receptor
SAM: *S*-Adenosylmethionine
Arg: L-arginine
Lys: L-lysine
IDO: Indoleamine 2,3-dioxygenase
ER: Endo-plasmic reticulum
HK1: Hexokinase 1
LDHA: Lactate dehydrogenase A
NPY: Neuropeptide Y
AgRP: Agouti-related protein
NAD: Nicotinamide adenine dinucleotide
HFD: Feeding high-fat diet
MTNR1B: Melatonin receptor 1B
TRAP: Tartrate-resistant acid phosphatase-5p
T2DM: Type 2 diabetes mellitus
DM: Diabetes mellitus
DIO: Diet-induced obesity
CREBH: CREB, hepatocyte specific
PRL: Phosphatase of regenerating liver
PPAR-2: Peroxisome proliferator-activated receptor
